# Nucleotide-dependent activities of FtsA regulate the early establishment of a functional divisome during the *Escherichia coli* cell cycle

**DOI:** 10.3389/fmicb.2023.1171376

**Published:** 2023-05-12

**Authors:** Josiah J. Morrison, Colby N. Ferreira, Evelyn M. Siler, Katie Nelson, Catherine E. Trebino, Benjamin Piraino, Jodi L. Camberg

**Affiliations:** Department of Cell and Molecular Biology, The University of Rhode Island, Kingston, RI, United States

**Keywords:** division, cytokinesis, actin, tubulation, Z-ring

## Abstract

During cell division in *Escherichia coli*, the highly conserved tubulin homolog FtsZ polymerizes and assembles into a ring-like structure, called the Z-ring, at the site of septation. For recruitment to the membrane surface, FtsZ polymers directly interact with membrane-associated proteins, predominantly FtsA in *E. coli*. FtsA shares structural homology with actin and, like actin, hydrolyzes ATP. Yeast actin detects nucleotide occupancy through a sensor region adjacent to the nucleotide binding site and adopts distinct conformations in monomeric and filamentous actin. Bacterial actin homologs also display considerable conformational flexibility across different nucleotide-bound states and polymerize. Here, we show that several amino acid residues proximal to the nucleotide binding site in FtsA are critical for function *in vitro* and *in vivo*. Each of these residues are important for ATP hydrolysis, phospholipid (PL) binding, ATP-dependent vesicle remodeling, and recruitment to the divisome *in vivo*, to varying degrees. Notably, we observed that Ser 84 and Glu 14 are essential for ATP-dependent vesicle remodeling and magnesium-dependent membrane release of FtsA from vesicles *in vitro*, and these defects likely underlie the loss of function by FtsA(E14R) and FtsA(S84L) *in vivo*. Finally, we demonstrate that FtsA(A188V), which is associated with temperature-sensitive growth *in vivo*, is defective for rapid ATP hydrolysis and ATP-dependent remodeling of PL vesicles *in vitro*. Together, our results show that loss of nucleotide-dependent activities by FtsA, such as ATP hydrolysis, membrane binding and release, and, most importantly, ATP-dependent PL remodeling, lead to failed Z-ring assembly and division defects in cells.

## Introduction

The bacterial cell cycle proceeds by duplicating the bacterial genome and then coordinating a division event to ensure that progeny cells inherit a complete copy of the chromosome. The physical process by which bacterial cells divide is widely conserved and essential and includes many proteins that collaborate to reshape and build new cell wall at the division septum. In *Escherichia coli*, the process of division and separation begins with the establishment of a network of cytoskeletal proteins, called the divisome, and the formation of a Z-ring. FtsZ polymers, containing protomers of the conserved, tubulin-like protein FtsZ, coalesce at midcell on the interior of the cell membrane and are recruited there by interactions with FtsA, an actin homolog, and ZipA to form the Z-ring (Pichoff and Lutkenhaus, 2002). FtsZ polymers are single stranded protofilaments that assemble head-to-tail by binding to GTP (Oliva et al., 2004). FtsZ polymers dissociate *in vitro* as GTP is hydrolyzed, and dynamic assembly, disassembly and reassembly is thought to be a key activity that contributes to promoting the division event *in vivo* (McQuillen and Xiao, 2020). FtsA, a bacterial cytoskeletal protein with homology to actin, recruits FtsZ to the phospholipid (PL) membrane and is also reported to dynamically assemble at the Z-ring along with FtsZ (Loose and Mitchison, 2014; Bisson-Filho et al., 2017; Conti et al., 2018; Caldas et al., 2019).

FtsA is a widely conserved member of the Hsc70/sugar kinase/actin family and is present in most bacteria (Bork et al., 1992). *E. coli* FtsA assembles into actin-like polymers, promoted by ATP binding (Szwedziak et al., 2012; Morrison et al., 2022), and the C-terminal region of FtsA intercalates into the PL membrane bilayer (Yim et al., 2000; Pichoff and Lutkenhaus, 2005; Conti et al., 2018). A truncated FtsA variant without the C-terminal 15 amino acid residues, FtsA(ΔMTS), polymerizes with ATP, indicating that PL binding is not required for polymerization; however, this variant weakly hydrolyzes ATP, in contrast to wild type FtsA’s highly processive ATPase activity in the presence of PL’s (Conti et al., 2018; Morrison et al., 2022). It is not understood how PL engagement coordinates or communicates with the active site of FtsA to regulate protein conformation or enzymatic activity, and further, how FtsA coordinates with FtsZ. When associated with PL vesicles, FtsA induces tubulation and vesicle remodeling when ATP is added, consistent with polymerization on the PL surface (Conti et al., 2018). When recruited to a supported lipid bilayer, FtsA assembly is surface restricted and can form PL-associated minirings that recruit FtsZ (Krupka et al., 2017). FtsA has also been shown to destabilize and remodel FtsZ polymers *in vitro* and one variant, FtsA(R286W), has been reported to require adenine nucleotide for FtsZ polymer destabilization (Beuria et al., 2009; Krupka et al., 2017; Conti et al., 2018).

To begin to address the interplay of ATP hydrolysis and function of FtsA, and protein communication between FtsA and FtsZ in the early steps of Z-ring assembly, we constructed mutations in the active site of FtsA and studied variant activity *in vitro* and *in vivo*. Specifically, we focused on residues near the site of magnesium coordination since magnesium is expected to be required for catalysis and may therefore be important in regulating nucleotide-dependent conformational transitions. Using a variety of biochemical and biophysical assays, we compared ATP hydrolysis, PL-dependent remodeling, ATP-dependent lipid tubulation, and FtsZ polymer destabilization by FtsA wild type and mutant proteins and correlated these *in vitro* activities with function and ring localization *in vivo*. Our results show that amino acid residues near the catalytic site are important for supporting and regulating FtsA function.

## Results

### Active site mutations in FtsA alter function *in vivo*, ATP hydrolysis, and PL binding

**FIGURE 1:**
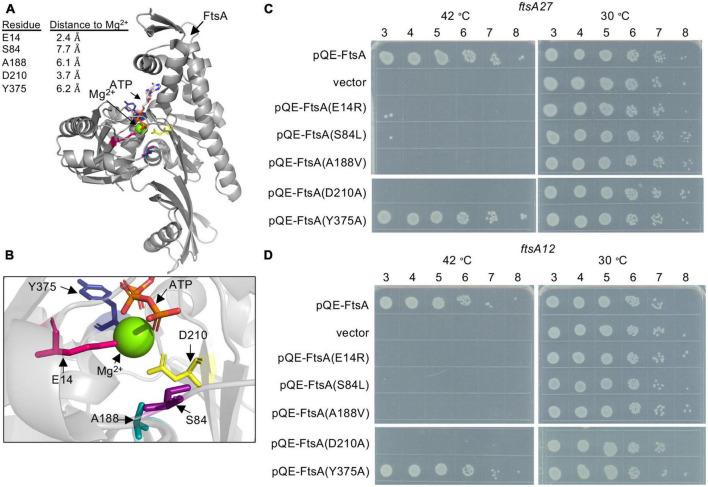
*Escherichia coli* FtsA active site amino acid residues are important for function. **(A)** *E. coli* FtsA (residues 1–405) (Nierhaus et al., 2022) (pdb: 7Q6D). *E. coli* amino acids Glu 14 (pink), Ser 84 (purple), Ala 188 (teal), Asp 210 (yellow) and Tyr 375 (blue) are shown as sticks. Distances from modeled residues to Mg^2+^ are indicated in angstroms (Å). **(B)** Enlarged view of *E. coli* FtsA active site in panel **(A)**. **(C)** Growth of *E. coli* strains containing expression plasmid pQE-9 (vector control), pQE9-*ftsA*, pQE9-*ftsA(E14R)*, pQE9*-ftsA(S84L)*, pQE9-*ftsA(A188V)*, pQE9-*ftsA(Y375A)*, or pQE9-*ftsA(D210A)* in MCA27 or **(D)** MCA12 temperature sensitive strains. Culture dilutions (1-log) were spotted (5 μl) on LB agar with ampicillin and grown as described in Section “Materials and methods”. Plates were incubated for 16 h at 30°C and 42°C. Data shown is representative of three replicates.

The FtsA active site is present at the center of the FtsA protomer, and accessibility to the central active site also appears to be maintained in actin-like filaments of *Thermotoga maritima* FtsA (van den Ent and Löwe, 2000; Szwedziak et al., 2012). To investigate active site mutations in FtsA, we selected amino acid residues near the corresponding active site *magnesium* in the structural model of *E. coli* FtsA (Figure 1A; Nierhaus et al., 2022) and performed site-directed mutagenesis to construct the following substitutions: E14R, S84L, A188V, D210A, and Y375A. Mutations were incorporated to modify the charge, polarity, or space occupancy of the amino acid side chains while minimally disrupting surrounding structures. Moreover, A188V is a previously reported mutation in FtsA that confers a thermosensitive growth defect (Herricks et al., 2014). All selected amino acid side chains are between 2 and 8 angstroms (Å) from the magnesium cation at the center of the *E. coli* FtsA active site in the available structure (Nierhaus et al., 2022; Figures 1A, B; Supplementary Figure 1A). Glu 14 (E14) and Asp 210 (D210) are situated in the phosphate-1 and phosphate-2 motifs, respectively (Bork et al., 1992), and thus predicted to be important for interacting with ATP phosphates and for hydrolysis activity. A recent report corroborated that FtsA(E14R) was defective for ATP hydrolysis *in vitro* and function *in vivo* (Morrison et al., 2022), providing a comparative basis to evaluate other mutations in this region of FtsA.

Many *ftsA* substitution mutations located proximal to the FtsA ATP binding pocket have been reported to confer temperature-sensitive growth in *E. coli* (Supplementary Figure 1A), including the temperature-sensitive strains, MCA27 (*ftsA*27) and MCA12 (*ftsA*12) (Dai et al., 1993). MCA27 contains a substitution mutation in chromosomal *ftsA* converting Ser 195 to Pro, and MCA12 contains a substitution mutation that converts Ala 188 to Val. In both of these strains, growth is prevented at high temperature (42°C), but the cells grow robustly at the permissive temperature (30°C). To compare function of FtsA mutant proteins by a complementation assay, cultures of MCA12 and MCA27 cells containing a control vector (pQE-9) or an expression vector containing wild type *ftsA* or *ftsA* variants were cultured, spotted onto LB agar plates containing ampicillin, and then grown overnight at 30 and 42°C. As expected, cells containing pQE-FtsA, containing the wild type *ftsA* gene, grew at the restrictive temperature, whereas cells containing the control vector did not (Figures 1C, D). Cells with plasmids containing four of the *ftsA* variants were unable to complement in either background at the restrictive temperature, including genes encoding FtsA(E14R), FtsA(S84L), FtsA(A188V), and FtsA(D210A) (Figures 1C, D). FtsA(A188V) contains a conservative mutation; however, it is the basis for the temperature-sensitive phenotype underlying MCA12. Our results demonstrate that plasmid encoded *ftsA(A188V)* is not sufficient to alleviate the temperature sensitive phenotype and suggest that the underlying defect may be related to a specific loss of FtsA function. Mutation of Ser 84 also led to loss of function, suggesting that this residue may provide an important contact or activity for FtsA. As expected, neither FtsA(E14R) nor FtsA(D210A) supported growth at the restrictive temperature in either strain background, consistent with previous reports of loss of function associated with mutation of these amino acids (Yim et al., 2000; Pichoff and Lutkenhaus, 2007; Lutkenhaus et al., 2012; Morrison et al., 2022).

**FIGURE 2:**
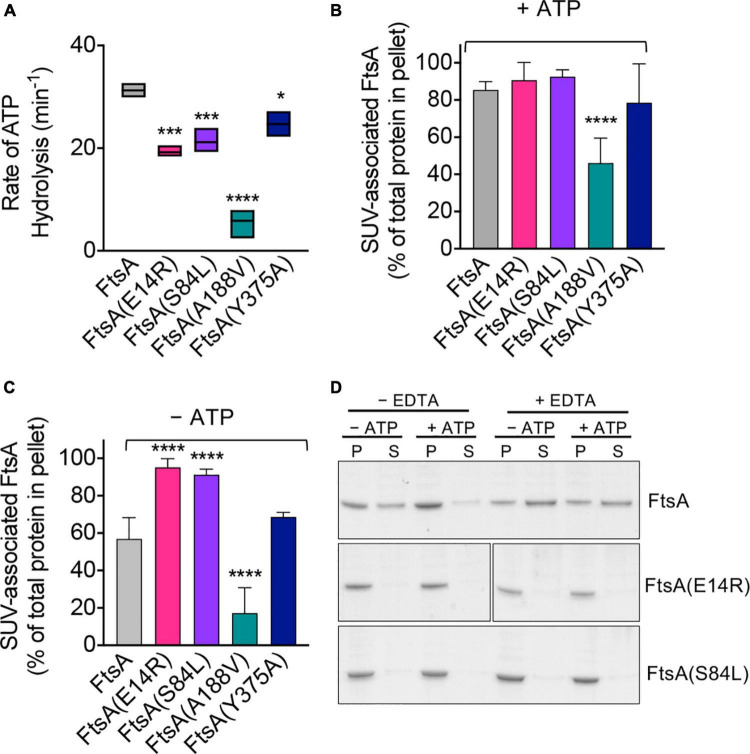
ATP hydrolysis and PL binding activity of FtsA wild type and mutant proteins. **(A)** ATP hydrolysis rates of FtsA wild type and FtsA mutant proteins (1 μM) were assayed by measuring the release of phosphate over time in reactions containing ATP (4 mM) as described in Section “Materials and methods”. Statistical analyses for data in **(A)** performed by comparison to FtsA wild type value (*p-value < 0.02; ***p-value < 0.001; ****p-value < 0.0001). **(B)** Recruitment of FtsA wild type and mutant proteins (1 μM) in assay buffer supplemented with SUV’s (250 μg ml^– 1^) with and without **(C)** ATP (4 mM) as described in Section “Materials and methods”. Data shown is an average of at least three replicates with error represented as S.E.M. Statistical analyses for data in **(B,C)** performed by comparison to FtsA wild type values (****p-value < 0.0001). **(D)** Phospholipid recruitment of FtsA, FtsA(E14R), and FtsA(S84L) (2 μM) to SUV’s (250 μg ml^– 1^) in the presence and absence of ATP (4 mM) and EDTA (25 mM) to chelate Mg^2+^ as described in Section “Materials and methods”. Data shown is representative of three replicates.

To gain further insight into how mutations of residues adjacent to the magnesium site correlate with the various activities of FtsA that have been reported *in vitro*, we overexpressed and purified each FtsA mutant protein from *E. coli* and characterized it for the ability to hydrolyze ATP and bind to PL’s. FtsA(E14R), FtsA(S84L), FtsA(A188V), and FtsA(Y375A) were purified as described for wild type FtsA (Supplementary Figure 1B; Conti et al., 2018; Morrison et al., 2022); however, we were unable to purify FtsA(D210A) and obtained no protein yield. Like wild type FtsA, all FtsA variants copurified with a small amount of PL’s, as has previously been described, although FtsA(A188V) copurified with significantly fewer PL’s (Supplementary Figure 1C; Conti et al., 2018; Morrison et al., 2022). All FtsA variants were assayed for their ability to hydrolyze ATP, and all were observed to be partially defective for ATP hydrolysis under the conditions tested, with FtsA(A188V) being the most severe and retaining only 19% of wild type activity (Figure 2A). FtsA(Y375A) was the least affected and retained 80% of wild type activity (Figure 2A). In agreement with a previous report, we observed that FtsA(E14R) retains 61% of ATPase activity compared to wild type FtsA (Morrison et al., 2022), and FtsA(S84L) retained 68% of wild type ATPase activity (Figure 2A). As FtsA(A188V) confers temperature-sensitive growth *in vivo* (Dai et al., 1993), we compared FtsA(A188V) ATP hydrolysis *in vitro* at 30°C and 42°C, which corresponds to the restrictive temperature for growth *in vivo*. However, we observed no further defect, or stimulation, of the rate of ATP hydrolysis by FtsA(A188V) as temperature increased and under the conditions tested (Supplementary Figure 2). The results suggest that the underlying cause of temperature-sensitive growth is not directly related to FtsA ATP hydrolysis rates.

Next, to determine if FtsA mutant proteins are defective for ATP hydrolysis due to a failure to bind PL’s, which is essential for rapid ATP turnover by wild type FtsA (Conti et al., 2018), we performed a PL recruitment assay by low-speed centrifugation in the presence of exogenous SUV’s (250 μg ml^–1^), which were added in excess to reactions to supplement copurifying PL’s (Supplementary Figure 1C), and then we collected PL associated proteins (Figures 2B, C). We observed that all FtsA mutant proteins fractionated with the PL pellet in the presence of ATP, and that only FtsA(A188V) was partially impaired for PL recruitment (Figure 2B). In the absence of ATP, 29% less wild type FtsA fractionated with the PL pellet; however, FtsA(E14R) and FtsA(S84L) remained robustly associated with the PL fraction (Figure 2C). In contrast to FtsA(E14R) and FtsA(S84L), FtsA(A188V) was poorly recruited to PL’s when ATP was omitted (Figure 2C), although it was recruited to a lesser extent than with ATP (Figure 2B). These results show that FtsA(A188V) is defective for binding to PL’s, compared to wild type FtsA, and that FtsA(E14R) and FtsA(S84L) bind to PL’s promiscuously without ATP.

FtsA(S84L) and FtsA(E14R) are partially defective for ATP hydrolysis (Figure 2A), showing 32% and 39% reduced rates of ATP hydrolysis compared to wild type FtsA, respectively. However, in the PL recruitment assay, both FtsA mutant proteins associated with PL’s similarly in the absence and presence of ATP, in contrast to wild type FtsA, which displays reduced PL association without ATP. One possibility for this is that FtsA(E14R) and FtsA(S84L) may persist in the PL-bound conformation regardless of nucleotide status. Glu 14 and Ser 84 are close to the predicted site of magnesium binding in FtsA (2.4 Å and 7.7 Å, respectively), (Figures 1A, B), therefore we hypothesized that magnesium occupancy at this site is involved in the conformational transitions between PL-associated and PL-released FtsA. To investigate this further, we repeated the PL recruitment assay with FtsA and ATP in the presence of EDTA to chelate the magnesium and observed that without magnesium 60% of FtsA remained soluble and not bound to PL’s when ATP was present, whereas less than 5% remained soluble and not bound to PL’s when ATP and magnesium were available (Figure 2D). This suggests that Mg^2+^ *and* ATP occupancy promote PL-binding by FtsA. Next, we tested if FtsA(E14R) and FtsA(S84L) were also released from PL’s when treated with EDTA but observed that both proteins remained associated with PL’s under all conditions tested (Figure 2D). This suggests that mutations in Glu 14 and Ser 84 disrupt the ability to support release from the membrane, suggesting that in the apo state, without Mg^2+^ and ATP, the FtsA mutant proteins persist in a PL-bound conformation *in vitro*.

**FIGURE 3:**
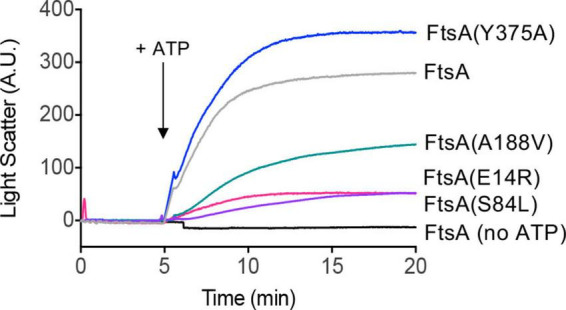
ATP-dependent PL remodeling by FtsA wild type and mutant proteins. FtsA wild type and FtsA mutant proteins (1 μM) were monitored by 90° angle light scatter at 450 nm. A baseline signal was measured for 5 min, ATP (4 mM) was added, and reactions were monitored for an additional 15 min. Data shown is representative of three replicates.

In previously reported light scattering assays (Conti et al., 2018; Morrison et al., 2022), FtsA was shown to remodel and tubulate PL vesicles when ATP is added to the reaction, and this activity likely corresponds to ATP-dependent polymerization *in vitro* (Morrison et al., 2022). To determine if FtsA mutant proteins are competent for tubulation of vesicles, we monitored 90° light scatter of reactions containing FtsA wild type and mutant proteins, and then added ATP to determine if ATP induces tubulation. In the absence of ATP, the light scatter signal produced by FtsA remained constant. We observed that wild type FtsA and FtsA(Y375A) both had a large increase in light scatter after ATP was added to the reactions (Figure 3); however, FtsA(E14R), FtsA(S84L), and, to a lesser extent, FtsA(A188V) were impaired for vesicle reorganization. The results suggest that while FtsA and FtsA(Y375A) are competent for ATP-induced vesicle remodeling, FtsA(E14R), FtsA(S84L), and FtsA(A188V) are defective.

**FIGURE 4:**
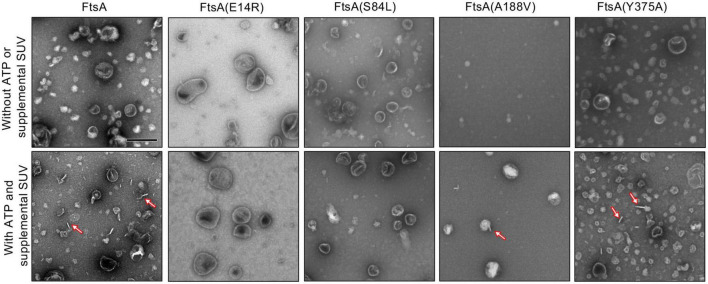
FtsA promotes vesicle tubulation with ATP. FtsA wild type and mutant proteins, FtsA(E14R), FtsA(S84L), FtsA(A188V), FtsA(Y375A), (4 μM) were incubated in the presence and absence of ATP (4 mM) and SUV’s (250 μg ml^– 1^) and visualized by TEM. Arrows indicate tubulated liposomes. Scale bar is 200 nm.

Finally, we visualized FtsA wild type and mutant proteins by negative stain transmission electron microscopy (TEM) with and without ATP and supplemental SUV’s to detect PL tubulation. We observed ATP-stimulated tubule formation with wild FtsA, FtsA(Y375A) and, to a lesser extent, FtsA(A188V), but failed to detect tubulation by FtsA(E14R) or FtsA(S84L) (Figure 4). The tubules observed were approximately 50−80 nm in length. These results are consistent with the reduced light scatter observed after addition of ATP by FtsA(E14R), FtsA(S84L), and to a lesser extent, FtsA(A188V), compared to wild type FtsA (Figure 3).

### FtsA mutant proteins are defective for destabilizing FtsZ polymers *in vitro*

**FIGURE 5:**
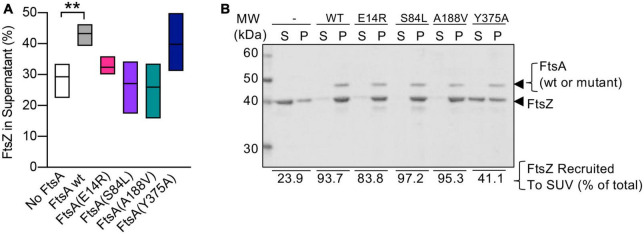
FtsA mutant proteins fail to destabilize FtsZ polymers. **(A)** Reactions containing FtsZ (3 μM), GTP (2 mM), and where indicated, FtsA, FtsA(E14R), FtsA(S84L), FtsA(A188V), FtsA(Y375A) (3 μM), and with ATP (4 mM) and a regenerating system containing acetyl phosphate (15 mM) and acetate kinase (25 μg ml^– 1^) were incubated for 5 min then fractionated by ultracentrifugation. Pellets and supernatants were visualized by SDS-PAGE and coomassie staining and quantified by densitometry. Data shown is an average of at least three replicates with error represented as S.E.M. Statistical analyses for data in **(A)** performed by comparison to “No FtsA” (**p-value = 0.0026). **(B)** Assays to recruit FtsZ to PL through an interaction with FtsA were performed by incubating FtsZ (3 μM) with GTP (2 mM) and then adding to a reaction containing FtsA wild type or mutant protein (2.5 μM), SUV’s (250 μg ml^– 1^), and ATP (4 mM) as described in Section “Materials and methods”. SUV’s and bound proteins were collected by centrifugation and analyzed by SDS-PAGE. Percent of FtsZ recruited to SUV’s is shown. Data shown is representative of three replicates.

FtsA binds to FtsZ polymers, recruits the polymers to PL’s, and promotes destabilization of polymerized FtsZ, releasing it into the soluble fraction while FtsA remains associated with PL’s (Conti et al., 2018; Morrison et al., 2022). To determine if FtsA mutant proteins are defective for disassembly of FtsZ polymers, we assembled FtsZ polymers with GTP, with and without FtsA and ATP, and then quantified soluble and polymerized fractions of FtsZ by SDS-PAGE (Figure 5A; Supplementary Figure 3). After incubation with GTP to induce polymerization of FtsZ, we observed that without FtsA and ATP, 29.3 ± 2.4% of FtsZ remained in the soluble, non-polymerized fraction after high-speed ultracentrifugation, and the majority of FtsZ sedimented as polymers (Figure 5A; Supplementary Figure 3). However, in the presence of FtsA and ATP, 43.3 ± 1.6% of FtsZ localized to the soluble fraction, suggesting that FtsA destabilizes FtsZ polymers and reduces the amount of FtsZ polymers collected by ultracentrifugation, consistent with destabilization or antagonizing activity by FtsA reported in other studies (Krupka et al., 2017; Conti et al., 2018; Morrison et al., 2022). The amount of FtsZ detected in the supernatant after incubation with FtsA(Y375A) was similar to wild type FtsA (39.8 ± 4.4%) (Figure 5A). However, we detected no increase in the amount of FtsZ detected in the supernatant after incubation with FtsA(E14R), FtsA(S84L), or FtsA(A188V), suggesting that the FtsA mutant proteins are defective for destabilizing FtsZ polymers under the conditions tested (Figure 5A).

Next, to determine if the failure to destabilize FtsZ polymers resulted from an inability of FtsA mutant proteins to interact with FtsZ, we performed a PL recruitment assay. In this assay, FtsZ polymers, assembled with GTP, were incubated with FtsA, ATP, and SUV’s, and then the SUV’s and bound proteins were collected by low-speed centrifugation and analyzed by SDS-PAGE. We observed that all FtsA mutant proteins were capable of recruiting FtsZ to varying degrees, including FtsA(S84L) and FtsA(A188V), and, both to a lesser extent, FtsA(E14R) and FtsA(Y375A) (Figure 5B). Together, these results suggest that FtsA mutant proteins FtsA(S84L) and FtsA(A188V), like FtsA(E14R), bind to FtsZ but are defective for destabilizing FtsZ polymers *in vitro*.

### FtsA mutant proteins are defective for ring assembly *in vivo*

**FIGURE 6:**
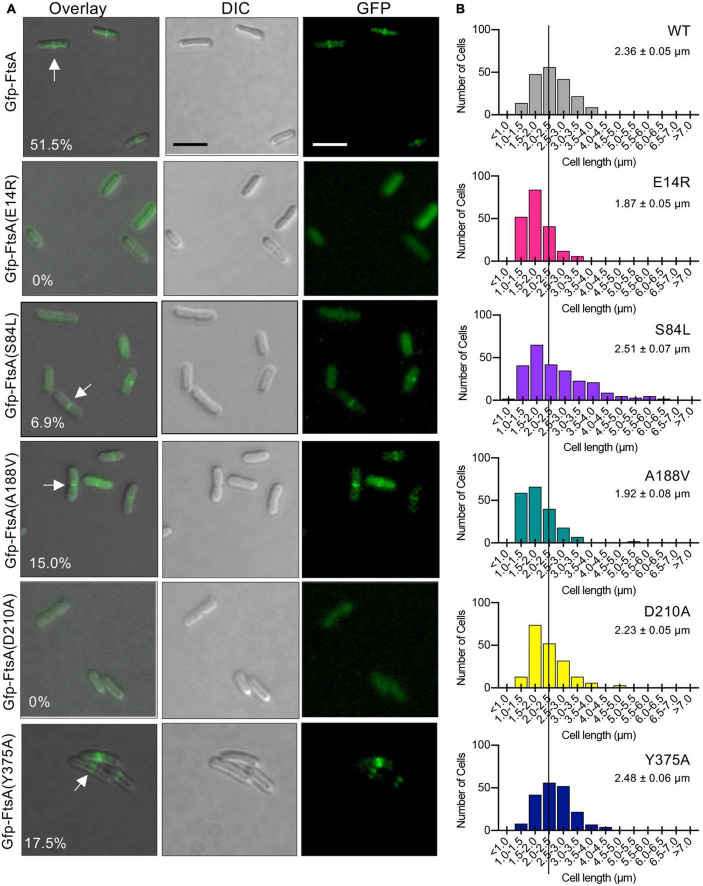
Fluorescence microscopy of Gfp-tagged FtsA mutant proteins. **(A)** Confocal fluorescence and differential interference contrast (DIC) microscopy of *E. coli* MG1655 *araE*_*CP*_ cells expressing plasmid-encoded Gfp-FtsA, Gfp-FtsA(E14R), Gfp-FtsA(S84L), Gfp-FtsA(A188V), Gfp-FtsA(D210A), or Gfp-FtsA(Y375A). Cells were grown at 30°C as described in Section “Materials and methods”. Scale bars are 2 μm. White arrows indicate A-rings. **(B)** Plots of cell lengths derived from cells grown as in panel **(A)**. Mean cell length is indicated for each mutant-expressing strain (S.E.M. is indicated) (*n* ≥ 200 cells for all strains).

To determine if FtsA mutant proteins that are defective for function *in vivo* in temperature-sensitive growth assays (Figures 1C, D) are also defective for localizing to a ring during division, we constructed and expressed each FtsA mutant protein as a fluorescent fusion protein to Gfp (green fluorescent protein) and imaged live dividing cells by fluorescence microscopy. We observed that expression of Gfp-FtsA from a plasmid [pSEB293; (Pichoff and Lutkenhaus, 2005)] led to the visualization of clear, robust rings (A-rings) at midcell in 51.5% of the population, and cells had a mean cell length of 2.36 ± 0.05 μm (Figures 6A, B). We also observed A-rings in cells expressing Gfp-FtsA(Y375A) (17.5%) and Gfp-FtsA(A188V) (15.0%), indicating that both mutant proteins are partially defective for A-ring formation. Cells expressing Gfp-FtsA(S84L), FtsA(D210A), or FtsA(E14R) had few to no visible rings (6.9%, 0% and 0%, respectively), and a mean cell length similar to cells expressing Gfp-FtsA (2.51 ± 0.07 μm, 2.23 ± 0.05 μm, and 2.48 ± 0.06 μm, respectively), (Figures 6A, B). However, cells expressing Gfp-FtsA(A188V) and Gfp-FtsA(E14R) were shorter than cells expressing Gfp-FtsA, with mean cell lengths of 1.92 ± 0.08 μm and 1.87 ± 0.05 μm, respectively. These results show that FtsA mutant proteins that are most defective for restoring growth of temperature-sensitive strains at the restrictive temperature [i.e., Gfp-FtsA(D210A), Gfp-FtsA(E14R), Gfp-FtsA(S84L), and Gfp-FtsA(A188V)] (Figures 1C, D) are also most defective for localizing to rings during division.

Finally, since FtsA(S84L) and FtsA(E14R) are defective for releasing from SUV’s *in vitro*, including with EDTA, we further tested if Gfp-FtsA(S84L) and Gfp-FtsA(E14R) demonstrate any visible membrane localization *in vivo*. We analyzed large populations of cells for Gfp-FtsA (wild type or mutant protein) localization and also stained the cells with the membrane dye FM 4−64. We failed to observe colocalization of either Gfp-FtsA(E14R) or Gfp-FtsA(S84L) with the bacterial membrane, and instead observed that both proteins were cytoplasmic, similar to Gfp without FtsA (Supplementary Figure 4), and, as reported above, incapable of localizing to a division ring.

## Discussion

Here, we report that FtsA mutant proteins with amino acid substitutions near the site of magnesium coordination in the active site of *E. coli* FtsA (Figures 1A, B) are associated with defects in supporting division *in vivo*, as well as defects in a variety of *in vitro* activities (Supplementary Table 1). We expressed FtsA mutant proteins from plasmids in two temperature sensitive strains carrying defective *ftsA* alleles on the chromosome to evaluate function *in vivo*. We observed that of the variants tested, only FtsA(Y375A) supported growth at the restrictive temperature at a level similar to cells carrying the wild type *ftsA* expression plasmid (Figures 1C, D). Thermosensitive strains would express both the mutant *ftsA* allele from the chromosome and a plasmid-encoded *ftsA* (wild type or variant). We expect that the variants constructed here are stably expressed, since fluorescence can be tracked when each variant is expressed as a Gfp fusion protein and Z-ring localization is quantified (Figure 6). The *in vitro* defect that most closely correlates with loss of function in this study is failure to promote ATP-dependent PL remodeling and tubulation, which is a measure of ATP-dependent polymerization of FtsA on the PL surface. It is expected that nucleotide *binding* would promote polymerization by FtsA, and that hydrolysis, supported by the active site magnesium, would therefore regulate how static or dynamic the polymers are by impacting nucleotide turnover (i.e., hydrolysis and release). Consistent with this, we previously reported that a truncated FtsA variant, without the C-terminal membrane targeting sequence (MTS), forms stable polymers *in vitro*, and the stable FtsA polymers bind to stable FtsZ polymers (Morrison et al., 2022), yet truncated FtsA fails to engage the PL membrane. This suggests that the ability of FtsA to oligomerize into polymers of undetermined length on the membrane is the critical feature for division that enables assembly of FtsA with FtsZ at a functional ring.

Interestingly, the *degree* of the ATP hydrolysis rate defect by each FtsA mutant protein does not closely correlate with the severity of functional defects *in vivo* (i.e., recruitment to division rings or restoring growth at the restrictive temperature in temperature sensitive strains) (Figures 1C, D, 6A). As an example, FtsA(A188V) does not hydrolyze ATP more rapidly at the permissive temperature relative to the restrictive temperature (Supplementary Figure 2). This result suggests that temperature sensitive defects mapping to *ftsA* mutations near the nucleotide binding site are suppressed by perhaps another division protein interaction affected by temperature downstream of FtsA. One possibility is that since *ftsA* temperature sensitive mutations are suppressed by changes to the FtsA protomer-protomer interface (Herricks et al., 2014), it is likely a protein that binds to FtsA in a conformation-dependent manner overcomes *ftsA* defects at low but not high temperature. Consistent with this, temperature-sensitive defects have also been mapped to *ftsK, ftsQ*, and *ftsI*, which can be overcome by FtsN multicopy expression, suggesting that FtsN recruitment and assembly may be the step responsive to thermal-sensitive regulation (Goehring et al., 2007). Importantly, FtsN also binds directly to FtsA (Baranova et al., 2020).

Based on the results reported here, purified wild type FtsA appears to be in an equilibrium between the PL-associated and unassociated states (Figures 2B, C), which can be shifted in response to ATP and magnesium, with amino acid residues in the magnesium proximal region being important determinants for the equilibrium, likely by stabilizing one or more conformations. Notably, FtsA(S84L) and FtsA(E14R) fail to polymerize and tubulate vesicles in light scattering assays and by electron microscopy, although they bind to PL’s (Figures 2B, C) and ATP *in vitro* (Figure 2A), suggesting that Ser 84 and Glu 14 stabilize a conformation relevant for division. Conversely, FtsA(A188V) is defective for binding to PL’s, compared to wild type FtsA, which may contribute to the slow rate of ATP hydrolysis observed (Figures 2A–C). Currently, significant structural information demonstrating various FtsA conformations is lacking; however, conformational flexibility and transitions largely drive differences in actin architecture (i.e., G-actin and F-actin), actin ATP hydrolysis rates, and protein-protein interactions. It is likely that the diverse array of biochemical and biophysical FtsA activities are all coordinately regulated with polymerization and protein-protein interactions, and that more needs to be understood about FtsA conformations to establish a detailed molecular model of FtsA’s precise activities during division. However, the capacity to polymerize at the membrane surface represents a critical feature of the role of FtsA in early division that is likely subject to regulation, similar to other cytoskeletal protein systems. This strategy ensures that proper Z-ring assembly itself serves as a critical checkpoint for cell cycle progression in bacteria.

## Materials and methods

### Bacterial strains and plasmids

Bacterial strains and plasmids used in this study are described in Table 1. Site-directed mutagenesis of *ftsA* was performed to construct substitution mutations S84L, A188V, D210A, and Y375A in pET-FtsA, pGfp-FtsA (pSEB293), and pQE9-FtsA (Pichoff and Lutkenhaus, 2005; Morrison et al., 2022).

**TABLE 1:** *E. coli* strains and plasmids.

Strain or plasmid	Relevant genotype	Source, references, or construction
**Strain**		
MG1655	LAM- -rph1	Blattner et al., 1997
MG1655 araECP	MG1655 (ΔaraEp kan PCP18-araE)	Viola et al., 2017
BL21 (ℷde3)	F- ompT gal dcm lon hsdSB(rB- mB-)	EMD Millipore, USA
	ℷ(de3[lacI lacUV5-T7 gene 1 Ind1 sam7 nin5])	
MCA12	F-, [araD139]B/r, leu-260:Tn10, ftsA12 (ts),	Dai et al., 1993
	Δ(argF-lac)169, λ-, e14-, flhD5301, Δ(fruK-yeiR)	
	725 (fruA25), relA1, rpsL150 (strR), rbsR22,	
	Δ(fimB-fimE)632 (:IS1), deoC1	
MCA27	F-, [araD139]B/r, leu-260:Tn10, ftsA27 (ts),	Dai et al., 1993
	Δ(argF-lac)169, λ-, e14-, flhD5301, Δ(fruK-yeiR)	
	725 (fruA25), relA1, rpsL150 (strR), rbsR22,	
	Δ(fimB-fimE)632 (:IS1), deoC1	
**Plasmid**		
pET-24b	kan, T7 promoter	EMD Millipore, USA
pET-ftsA	kan	Conti et al., 2018
pET-ftsA(E14R)	kan	Morrison et al., 2022
pET-ftsA(S84L)	kan	This study
pET-ftsA(A188V)	kan	This study
pET-ftsA(D210A)	kan	This study
pET-ftsA(Y375A)	kan	This study
pET-ftsZ	kan	Camberg et al., 2009
pSEB293	amp, gfp-ftsA in pBad18	Pichoff and Lutkenhaus, 2005, 2007
pBAD-gfp-ftsA(E14R)	amp, gfp-ftsA(E14R)	Morrison et al., 2022
pBAD-gfp-ftsA(S84L)	amp, gfp-ftsA(S84L)	This study
pBAD-gfp-ftsA(A188V)	amp, gfp-ftsA(A188V)	This study
pBAD-gfp-ftsA(D210A)	amp, gfp-ftsA(D210A)	This study
pBAD-gfp-ftsA(Y375A)	amp, gfp-ftsA(Y375A)	This study
pBAD-gfp(uv)	amp, gfp(uv)	Hoskins et al., 2000
pQE-9	amp T5 promoter	Qiagen, USA, and Meyer et al., 2000
pQE9-ftsA	amp	Morrison et al., 2022
pQE9-ftsA(E14R)	amp	Morrison et al., 2022
pQE9-ftsA(S84L)	amp	This study
pQE9-ftsA(A188V)	amp	This study
pQE9-ftsA(D210A)	amp	This study
pQE9-ftsA(Y375A)	amp	This study

### Temperature sensitive growth assays

*Escherichia coli* strains containing expression plasmid pQE-9, pQE9-*ftsA*, pQE9-*ftsA(E14R)*, pQE9*-ftsA(S84L)*, pQE9-*ftsA(A188V)*, pQE9-*ftsA(Y375A)*, or pQE9-*ftsA(D210A)* in temperature sensitive strains MCA27 [*ftsA*(S195P)] or MCA12 [*ftsA*(A188V)] were grown overnight in Lennox broth supplemented with 100 μg ml^–1^ ampicillin at 30°C. The following day, cultures were diluted into fresh LB with ampicillin to an OD_600_ of 0.05 and grown to an OD_600_ of 0.4 at 30°C shaking (250 rpm). Cultures were diluted (2-log) into LB containing ampicillin and spotted (5 μl) onto LB agar plates with ampicillin and incubated at 30°C (permissive) or 42°C (restrictive) for 16 h. Leaky expression of FtsA from the T5 promoter of pQE-*ftsA* is sufficient to support growth without inducer.

### Protein purification

FtsA wild type and mutant proteins FtsA(E14R), FtsA(S84L), FtsA(A188V), FtsA(D210A), and FtsA(Y375A) were expressed in cells induced with isopropyl β-d-1-thiogalactopyranoside (0.5 mM), and cultures were grown at 16°C for 20 h (Conti et al., 2018). FtsA wild type and mutant proteins were purified from soluble cell lysate by ammonium sulfate fractionation, anion exchange, and size exclusion chromatography as described (Conti et al., 2018). FtsZ was purified as described (Camberg et al., 2009). Protein concentrations refer to FtsA monomers and FtsZ monomers.

### Phospholipid assays

Phospholipid recruitment assays (25 μl) with FtsA wild type or mutant proteins containing substitutions (E14R), (A188V), (S84L), or (Y375A) were performed by incubating FtsA wild type or mutant proteins (1 μM) in 50 mM Tris pH 7.5, 150 mM KCl and 10 mM MgCl_2_ in the presence or absence of small unilamellar vesicles (SUV’s) (250 μg ml^–1^) with or without ATP (4 mM), or in the presence or absence of 25 mM EDTA where indicated, for 10 min at 30°C followed by 21,000 x g centrifugation for 15 min at 23°C. SUV’s were prepared from *E. coli* total membrane PL extracts (Avanti Polar Lipids) as described (Conti et al., 2018). Supernatants and pellets were analyzed by SDS-PAGE and coomassie staining. Percent of protein in the pellet fraction vs. supernatant fraction was determined by densitometry using NIH ImageJ. Where indicated, lipid concentrations of FtsA fractions were determined by incubation of FtsA with FM 4-64FX (1.25 μg ml^–1^) (Invitrogen). Fluorescence intensity was measured with an Agilent Eclipse spectrofluorometer using an excitation wavelength of 565 nm and an emission wavelength of 745 nm (slit widths 5/10 nm). Concentration was determined by comparing to a PL standard curve.

Recruitment assays with FtsA and FtsZ were performed by incubating FtsZ (3 μM) with GTP (2 mM), in reaction buffer for 2 min, and then adding to a reaction containing FtsA wild type or mutant protein (2.5 μM), as indicated, pre-assembled with SUV’s (250 μg ml^–1^) and ATP (4 mM). Reactions were incubated for an additional 10 min at 30°C, and then phospholipid vesicles were collected by low-speed centrifugation at 21,000 × *g* for 15 min. Supernatants and pellets were analyzed by SDS-PAGE and coomassie staining. Percent of protein in the supernatant fraction was determined by densitometry using NIH ImageJ.

To monitor ATP-dependent vesicle remodeling by light scatter, reaction mixtures (80 μl) containing reaction buffer and FtsA, FtsA(E14R), FtsA(S84L), FtsA(A188V), or FtsA(Y375A) (each at 1 μM) were monitored at 23°C for 5 min to collect a baseline then ATP (4 mM) was added, where indicated, and reactions were monitored for an additional 15 min at on an Agilent Eclipse spectrofluorometer 450 nm with 5 nm slit widths.

### ATP hydrolysis

ATP hydrolysis was measured by monitoring the amount of inorganic phosphate released at indicated temperatures in reactions (25 μl) using malachite green reagent (Enzo Life Sciences) for phosphate detection and comparing to a phosphate standard curve. ATP hydrolysis assays were performed at 37°C unless otherwise indicated in reaction buffer containing 50 mM Tris (pH 7.5), 150 mM KCl, and 10 mM MgCl_2_, ATP (4 mM) and FtsA (1 μM), or FtsA mutants (1 μM). Where indicated, SUV’s (250 μg ml^–1^) were added to reactions.

### Transmission electron microscopy

Reaction buffer containing either FtsA, FtsA(E14R), FtsA(S84L), FtsA(A188V), or FtsA(Y375A) (each 4 μM), with or without ATP (4 mM) and SUV’s (250 μg ml^–1^), where indicated, were incubated for 10 min at 23°C, applied to a 300-mesh carbon/formvar coated grid, fixed with glutaraldehyde (2.5%) and negatively stained with uranyl acetate (2%). Samples were imaged by transmission electron microscopy (TEM) using a JEM-2100 80 Kev instrument.

### FtsZ assembly assays

To collect FtsZ polymers by ultracentrifugation, reaction mixtures (25 μl) with FtsZ (3 μM) were prepared in reaction buffer containing 50 mM MES (pH 6.5), 100 mM KCl, and 10 mM MgCl_2_, 2 mM GTP and a nucleotide regenerating system containing acetyl phosphate (15 mM) and acetate kinase (25 μg ml^–1^). Where indicated, FtsA or FtsA mutant proteins (3 μM) and ATP (4 mM) were added. Reactions were incubated for 5 min at 23°C and then centrifuged at 160,000 x *g* for 30 min in a Beckman TLA 120.1 rotor. Pellets and supernatants were resuspended in equal volumes, analyzed by SDS-PAGE and Coomassie staining, and quantified by densitometry using NIH ImageJ.

### Fluorescence microscopy

Cultures of MG1655 *araE*_CP_ containing the plasmid pSEB293 (Pichoff and Lutkenhaus, 2005) encoding GFP-FtsA, Gfp-FtsA(E14R), Gfp-FtsA(S84L), Gfp-FtsA(A188V), Gfp-FtsA(D210A), and Gfp-FtsA(Y375A) were grown overnight on solid LB Lennox media containing ampicillin (100 μg ml^–1^) without arabinose (leaky expression was sufficient to observe localization) at 30°C and then streaked onto plates of the same composition and grown for 5 h at 30°C. Cells were collected and resuspended in 1x phosphate buffered saline (PBS) and, where indicated, incubated for 10 min in the dark at room temperature with FM 4−64FX (3 μg ml^–1^) (ThermoFisher). Cells were applied to a 5% agarose pad containing M9 minimal media with glucose (0.4%), and then a coverslip was added. Samples were visualized with a Zeiss LSM 700 confocal fluorescence microscope with excitation at 488 nm and emission at 555 nm for Gfp and 565 nm and 744 nm for FM 4−64FX, respectively. Where indicated, a Nomarski prism was used to acquire differential interference contrast (DIC) images. All images were captured on an AxioCam digital camera with ZEN 2012 software. Cell lengths were measured using NIH ImageJ.

## Data availability statement

The raw data supporting the conclusions of this article will be made available by the authors, without undue reservation.

## Author contributions

JM, ES, and JC designed the study. JM, CF, ES, KN, CT, and BP performed experiments. JM, CF, CT, and BP edited the manuscript. JM and JC wrote the manuscript. JC secured funding for the study. All authors contributed to the article and approved the submitted version.
